# Lack of association between p53 gene polymorphisms and primary open angle glaucoma in the Japanese population

**Published:** 2009-05-20

**Authors:** Fumihiko Mabuchi, Yoichi Sakurada, Kenji Kashiwagi, Zentaro Yamagata, Hiroyuki Iijima, Shigeo Tsukahara

**Affiliations:** 1 Department of Ophthalmology, Faculty of Medicine, University of Yamanashi, Yamanashi, Japan; 2 Department of Health Sciences, Faculty of Medicine, University of Yamanashi, Yamanashi, Japan

## Abstract

**Purpose:**

To assess whether tumor protein p53 gene (*p53*) polymorphisms are associated with primary open angle glaucoma (POAG) in the Japanese population.

**Methods:**

Four hundred and twenty-five Japanese patients with POAG, including normal tension glaucoma (NTG, n=213) and high tension glaucoma (HTG, n=212) and 189 control subjects without glaucoma were analyzed for two *p53* polymorphisms (rs1042522; a G→C substitution at codon 72 in exon 4 and rs59758982; a 16 base pair insertion in intron 3) using allele specific primer PCR and a pyrosequencing technique respectively. The genotypic and allelic frequencies were compared between NTG or HTG patients and control subjects.

**Results:**

No significant difference (NTG versus control, p=0.99, and HTG versus control, p=0.69, χ^2^ test) was observed regarding the p53 genotype frequencies at codon 72 between the NTG (GG: 43.2%, GC: 44.6%, CC: 12.2%) or HTG (GG: 40.1%, GC: 48.1%, CC: 11.8%) patients and the control subjects (GG: 43.9%, GC: 43.9%, CC: 12.2%). In addition, there was no significant difference (NTG versus control, p=0.94; and HTG versus control, p=0.66, Fisher's exact test) in the *p53* allele frequencies at codon 72 between the NTG (G allele: 65.5%, C allele: 34.5%) or HTG (G allele: 64.2%, C allele: 35.8%) patients and the control subjects (G allele: 65.9%, C allele: 34.1%). No 16 base pair insertion in intron 3 was found in this study.

**Conclusion:**

*p53* polymorphisms were not associated with POAG in the Japanese population. Further studies in the other ethnic populations should therefore be performed to elucidate whether the *p53* intron 3 insertion polymorphism is a genetic risk factor for POAG, because the intron 3 insertion polymorphism occurs very rarely in the Japanese population.

## Introduction

Glaucoma is characterized by optic nerve atrophy with an excavated optic nerve head and progressive visual field loss, and it can potentially result in bilateral blindness. Primary open-angle glaucoma (POAG) is the most common form of glaucoma, and it is clinically classified into high tension glaucoma (HTG), in which elevated intraocular pressure (IOP) is a major feature, and normal tension glaucoma (NTG), in which the IOPs are consistently within the statistically normal population range. Although an elevation of IOP is recognized as a major risk factor for optic nerve damage in POAG [1], POAG is a complex and genetically heterogeneous disease characterized by the progressive apoptotic death of retinal ganglion cell (RGC), and apoptosis-related genes seem to possibly be associated with POAG, especially NTG.

Tumor protein p53 (p53) is a nuclear phosphoprotein key regulator of the cell cycle, and this protein is essential for cell cycle arrest and the induction of apoptosis. Previous studies have shown p53-associated apoptosis to be involved in POAG pathogenesis [2], and p53 gene polymorphism may contribute to the pathogenesis of POAG. In 2002, Lin et al. [3] first reported the *p53* codon 72 polymorphism to be associated with POAG. However, the association between such polymorphisms in *p53* and POAG remains controversial according to previous reports [3-6]. Further studies with a larger number of cases and different ethnic populations are thus called for to more fully elucidate the relationship between *p53* and POAG. In this study, we investigated whether polymorphisms in *p53* were associated with POAG in the Japanese population.

## Methods

### Subjects

Japanese patients with POAG and control subjects were recruited from ophthalmology practices in University of Yamanashi Hospital, Enzan Municipal Hospital, Uenohara City Hospital, and Oizumi Clinic in Yamanashi or Nagano prefectures, Japan. A diagnosis of POAG was made when open angles were detected on a gonioscopic examination and typical glaucomatous cupping of the optic disc (thinning of the optic disc rim and/or enlargement of the horizontal optic disc cupping) with compatible visual field defects (nasal step and/or partial arcuate visual field defect) was detected by automated static perimetry (Humphrey Visual Field Analyzer 30-2, Humphrey Instruments, San Leandro, CA). In patients with POAG, patients with HTG had evidence of at least one previous IOP measurement that was more than 21 mm Hg using a Goldmann applanation tomometer. In contrast, patients with NTG showed an IOP of less than 21 mm Hg at all tests. The patients were excluded if they had a history of eye surgery, including laser treatment, before the diagnosis of POAG. The control subjects consisted of Japanese who were over 40 years of age, had IOP below 21 mm Hg, exhibited no glaucomatous cupping of the optic disc (no thinning of disc rim and cup-to-disc ratio less than 0.4) and had no family history of glaucoma. All participants received comprehensive ophthalmologic examinations, and peripheral blood was collected. The study protocol was approved by the Ethics Committee of University of Yamanashi and informed consent was obtained from all study participants. The study was conducted in accordance with the Declaration of Helsinki.

### Genomic DNA genotyping

Genomic DNA was purified with a Flexi Gene® DNA Kit (Qiagen, Valencia, CA) and two *p53* polymorphisms, a G→C substitution at codon 72 in exon 4 (rs1042522) and a 16 base pair insertion in intron 3 (rs59758982), were genotyped. An allele specific primer PCR method was used for genotyping rs1042522, and the primers shown in Table 1 were used for amplification. The 1st PCRs were carried out in a total volume of 10 μl containing 10 ng genomic DNA, 0.5 μM of each primer, 200 μM of each dNTP, 1.5 mM of MgCl_2_, and 0.5 U of Taq polymerase (GoTaq® Flexi DNA polymerase; Promega Corporation, Madison, WI) in a thermocycler (GeneAmp PCR System 9700; Applied Biosystems Inc, Foster City, CA). Amplification was carried out with an initial denaturation at 94 °C for 2 min, followed by 35 cycles of denaturation at 94 °C for 20 s, annealing at 55 °C for 30 s, and extension at 72 °C for 30 s. A final extension at 72 °C for 7 min completed the reactions. The 2nd PCRs were carried out in a total volume of 20 μl containing 1st PCR product, 0.2 μM of each primer, 200 μM of each dNTP, 1.5 mM of MgCl_2_, and 0.5 U of Taq polymerase (GoTaq® Flexi DNA polymerase; Promega Corporation) in a thermocycler (GeneAmp PCR System 9700; Applied Biosystems Inc). Amplifications for G and C allele genotyping were carried out with an initial denaturation at 94 °C for 2 min, followed by 15 cycles of denaturation at 94 °C for 20 s and annealing at 65 °C and 62 °C for 30 s, respectively. The amplification products were electrophoresed with 2% agarose gels, and genotypes were obtained. To perform accurate genotyping, the amplification products were accurately genotyped by direct sequencing, and then were used as positive controls for this method. A pyrosequencing analysis was performed for genotyping rs59758982 as previously described [7]. The primers shown in Table 1 were used for amplification and sequencing. The 5-end biotinylation for the PCR reverse primer was performed.

**Table 1:** The primers used in this study.

**Allele specific primer PCR method**	**Annealing temperature**	**Product size**
1st PCR		
Forward Primer: 5’-TGGTCCTCTGACTGCTCTTT-3’	55 °C	531 bp
Reverse Primer: 5’-AGGGTGTGATGGGATGGATAA-3’		

2nd PCR for G allele genotyping		
Forward Primer: 5’-ATGCCAGAGGCTGCTCGCCG-3’	65 °C	398 bp
Reverse Primer: 5’-AGGGTGTGATGGGATGGATAA-3’		

2nd PCR for C allele genotyping		
Forward Primer: 5’-TGGTCCTCTGACTGCTCTTT-3’	62 °C	172 bp
Reverse Primer: 5’-TGCTGGTGCAGGGGCCTCGG-3’		

**Pyrosequencing analysis**		
PCR forward primer: 5’-AATTCCATGGGACTGACTTTCTGC-3’	60 °C	370 bp
PCR reverse primer: biotin labeled 5’-Bio-GGGAAGGGACAGAAGATGACAG-3’		
Sequencing reverse primer: 5’-GGACAAGGGTTGGGC-3’		

### Statistical analysis

Data were analyzed using SAS statistical software (version 9.1, SAS Institute Inc., Cary, NC). A χ^2^ analysis of the Hardy-Weinberg equilibrium for *p53* genotypes was performed for the patients and controls. Genotypic and allelic frequency differences between POAG patients and control subjects were estimated by the χ^2^ test and Fisher’s exact test, respectively. A value of p<0.05 was considered to be statistically significant.

## Results

Four hundred and twenty-five Japanese patients with POAG (213 patients with NTG and 212 patients with HTG) and 189 control subjects were enrolled in this study. The demographic and clinical data in the patients with POAG and the control subjects are shown in Table 2. The mean age at the time of blood sampling was 63.4±14.3 years (standard deviation) in patients with POAG and 65.5±11.4 years in the control subjects. The mean age at the time of diagnosis was 57.4±13.3 and 54.4±15.3 years in patients with NTG and HTG, respectively. The mean of maximum known IOP was 23.5±8.2 in patients with POAG and 15.0±2.7 mmHg in the control subjects.

**Table 2:** Demographic and clinical features in control subjects and patients with POAG.

**Demographic and clinical features**	**Control** **(n = 189)**	**POAG**	
		**NTG** **(n = 213)**	**HTG** **(n = 212)**
Age at blood sampling (years)	65.5±11.4	63.9±13.7	62.9±14.8
Age at diagnosis (years)	-	57.4±13.3	54.4±15.3
Male Gender, n (%)	70 (37.0)	91 (42.7)	129 (60.8)
Maximum IOP (mmHg)	15.0±2.7	18.5±1.9	28.5±9.0
Refractive error (diopter)	-0.4±2.2	-2.0±3.4	-2.1±3.0
Familial history of glaucoma, n (%)	0 (0)	48 (22.5)	62 (29.2)

POAG: primary open angle glaucoma, NTG: normal tension glaucoma, HTG: high tension glaucoma, IOP: intraocular pressure. Continuous variables are expressed as mean±standard deviation.

The genotype and allele frequencies of *p53* polymorphisms (rs1042522 and rs59758982) in patients with POAG and the control subjects are shown in Table 3. The *p53* genotype and allele frequencies were in Hardy-Weinberg equilibrium in patients with NTG and HTG and the control subjects. No significant difference (NTG versus control, p=0.99, and HTG versus control, p=0.69, χ^2^ test) was observed regarding the *p53* genotype frequencies at codon 72 (rs1042522) between the NTG (GG: 43.2%, GC: 44.6%, CC: 12.2%) or HTG (GG: 40.1%, GC: 48.1%, CC: 11.8%) patients and the control subjects (GG: 43.9%, GC: 43.9%, CC: 12.2%). In addition, no significant difference (NTG versus control, p=0.94; and HTG versus control, p=0.66, Fisher's exact test) was observed in the *p53* allele frequencies at codon 72 between the NTG (G allele: 65.5%, C allele: 34.5%) or HTG (G allele: 64.2%, C allele: 35.8%) patients and the control subjects (G allele: 65.9%, C allele: 34.1%). No 16 base pair insertion in intron 3 (rs59758982) was found.

**Table 3:** Genotype and allele frequencies of rs1042522 and rs59758982 of tumor protein p53 (*p53*) in patients with POAG and control subjects.

***p53* polymorphisms** **(rs number)**		**Control (n=189)**	**POAG**			
			**NTG**		**HTG**	
			**(n=213)**	**p value**	**(n=212)**	**p value**
rs1042522 (Arg72Pro)	Genotype					
	CC	23 (12.2)	26 (12.2)		25 (11.8)	
	CG	83 (43.9)	95 (44.6)	0.99*	102 (48.1)	0.69*
	GG	83 (43.9)	92 (43.2)		85 (40.1)	
	Allele					
	C	129 (34.1)	147 (34.5)		152 (35.8)	
	G	249 (65.9)	279 (65.5)	0.94**	272 (64.2)	0.66**
rs59758982 (16 bp intron 3 insertion)	Genotype					
	Insertion/ Insertion	0 (0)	0 (0)		0 (0)	
	Insertion/ Deletion	0 (0)	0 (0)	NA	0 (0)	NA
	Deletion/ Deletion	189 (100)	213 (100)		212 (100)	
	Allele					
	Insertion	0 (0)	0 (0)		0 (0)	
	Deletion	378 (100)	426 (100)	> 0.99**	424 (100)	> 0.99**

POAG: primary open angle glaucoma, NTG: normal tension glaucoma, HTG: high tension glaucoma, Arg: arginine, Pro: proline, NA: not available. Data are given as numbers (percentage). The asterisk indicates that the data were analyzed by a χ^2^ test and the double asterisk indicates that the Fisher's exact test was used.

## Discussion

In the current study, no significant differences were found between the genotype and the allele frequencies of the *p53* codon 72 polymorphism (rs1042522) among HTG or NTG patients and control subjects. The statistical power was above 94% to detect any significant difference of the allele frequencies among the diagnostic groups at α=0.05. This result contrasts with that of Lin et al. [3], who found a significantly higher prevalence of minor C allele at codon 72 G/C polymorphism caused the amino acid alteration from arginine to proline in the 58 Chinese patients with POAG in comparison to the 59 control subjects, although it is mostly the same as those of the other previous studies [4-6]. There are several possible explanations for this discrepancy. The finding of Lin et al. [3] may thus have possibly been false positive because of their small cohort size. The Arg72 variant has been shown to induce apoptosis markedly better than does the Pro72 variant [8]. Therefore, the Arg72 polymorphism may be biologically expected to confer apoptotic susceptibility. However, Lin et al. [3] concluded that the Pro72, not the Arg72 variant, was a significant risk factor for POAG. An alternative explanation may be a different case definition of the patients in each study. A variety of genetic factors may therefore contribute to optic neuropathy in POAG, and such factors may thus be classified to IOP and non-IOP related genetic factors. Although it is not yet clear how *p53* polymorphisms might act, it is presumed that *p53* contributes to optic neuropathy as a non-IOP related genetic factor, because p53-associated apoptosis of RGC seems to be involved in POAG pathogenesis [2]. Based on this point of view, we classified the POAG patients as two groups (NTG and HTG) by the IOP to evaluate the allele and genotype frequencies, because the non-IOP related genetic factors would predominate in patients with NTG while, on the contrary, the high IOP related genetic factors would predominate in patients with HTG. On the other hand, their study did not classify the POAG patients by the IOP. Alternatively, this different result between the Chinese and Japanese population might be due to either genetic heterogeneity or the different genetic pathogenesis of POAG between the two ethnic groups.

As for 16 base pair insertion polymorphism (rs59758982) in intron 3, Ressiniotis et al. [5] reported a significant difference in the *p53* haplotype (rs1042522 and rs59758982) distribution between 140 British patients with POAG and 73 control subjects, and thus described that for individuals with 16 base pair insertion polymorphism an Arg72 was significantly more common in the patients than the controls. Acharya et al. [4] reported that the allele containing the 16 base pair insertion was under represented (p=0.028) in 67 Indian patients with POAG compared to that in 112 control subjects, although no significant (p=0.059) genotype frequency difference was found between them. The *p53* intron 3 insertion or this insertion combined with an Arg72 polymorphism may contribute to optic neuropathy for POAG. Dimasi et al. [6] reported no significant difference in the same haplotype distribution between 283 Australian HTG or 62 NTG patients and 178 control subjects. However, the cohort size of NTG may not have been sufficient, as they mentioned in their study [6]. Interestingly, no 16 base pair insertion in intron 3 was found in this study. Hiyama et al. [9] also described this insertion polymorphism to be a rare event in the Japanese population. The reason for dissimilar findings may reflect the ethnic difference in the *p53* intron 3 insertion allele frequencies. At least, the POAG patients associated with the intron 3 insertion or this insertion combined with an Arg72 polymorphism are very few in the Japanese population, even though these polymorphisms would contribute to optic neuropathy in POAG.

In conclusion, *p53* polymorphisms were not associated with POAG in the Japanese population. Further studies in other ethnic populations with large numbers of patients and well-defined diagnostic criteria should thus be performed to elucidate whether the *p53* intron 3 insertion or this insertion combined with a codon 72 polymorphism is a genetic risk factor for POAG, especially NTG, because the intron 3 insertion polymorphism seems to be a rare event in the Japanese population.
